# Association of body mass index and height with risk of prostate cancer among middle-aged Japanese men

**DOI:** 10.1038/sj.bjc.6602983

**Published:** 2006-02-07

**Authors:** N Kurahashi, M Iwasaki, S Sasazuki, T Otani, M Inoue, S Tsugane

**Affiliations:** 1 Epidemiology and Prevention Division, Research Center for Cancer Prevention and Screening, National Cancer Center, 5-1-1 Tsukiji, Chuo-ku, Tokyo 104-0045, Japan

**Keywords:** prostate cancer, body mass index, body height, prospective study

## Abstract

In a population-based prospective study of 49 850 Japanese men, body mass index and height were not significantly associated with risk of prostate cancer (311 cases), although small positive effects could not be ruled out in advanced cases (91 cases).

The prostate cancer rate in Japan is much lower than in Western populations (Ajiki *et al*, 2004), but for reasons that are unclear. Endogenous hormones such as testosterone and insulin-like growth factor-I (IGF-I) are associated with prostate cancer (Kaaks *et al*, 2000), and also with height and body mass index (BMI). Studies of prostate cancer risk in relation to BMI and height have been inconsistent (IARC, 2002), partly because of differences in the range of BMI and height values used. One reason for the low incidence of prostate cancer in Japan may be the small proportion of tall men and men with a high BMI.

We conducted a population-based prospective study of BMI and height in relation to the risk of prostate cancer in Japanese men.

## MATERIALS AND METHODS

The Japan Public Health Centre-based Prospective Study (JPHC study), initiated in 1990 for Cohort I and in 1993 for Cohort II, has been described in detail previously (Otani *et al*, 2005). Cohort I included those aged 40–59 years resident in four public health centre (PHC) areas, and Cohort II those aged 40–69 years resident in six PHC areas. We enrolled men who provided valid responses to a self-administered questionnaire (77%), and excluded men with history of prostate cancer (*n*=3). Finally, a population-based cohort of 49 850 men was established.

The self-administered questionnaire asked about current height, body weight (thereby providing BMI), various lifestyle factors and medical history. We examined the correlation between data recorded in health check-ups and self-reported data. Spearman's correlation coefficients were 0.93 for height and 0.91 for BMI, respectively; the self-reported data were considered appropriate for use in the present study.

Subjects were followed from the baseline survey until 31 December 2003, during which time a total of 311 newly diagnosed prostate cancer cases were identified. Eight per cent of the subjects moved out of a study area and 0.2% were lost to follow-up during the study period.

The relative risk (RR) and 95% confidence interval (CI) of prostate cancer according to BMI and height were calculated by the Cox proportional hazards model, adjusting for potential confounders. We conducted additional analyses limited to the 91 advanced cases, with extraprostatic or metastatic spread. Of the remaining cases, 179 were organ localised and 41 (13.2% of total) were of undetermined stage.

## RESULTS

Body mass index and height were higher in young and married men than in others. Although the proportion of current smokers decreased with increasing BMI, it positively increased with increasing height. A family history of prostate cancer was not associated with BMI or height.

Table 1 presents RRs in relation to BMI and height for total cases and for each stage of diagnosis. Weak associations were found between BMI and total prostate cancer (RR=1.31 95% CI=0.97–1.76 for ⩾25 *vs* ⩽21.9, *P* for trend=0.057). However, multivariate RRs were attenuated (*P* for trend=0.13). When we analysed according to stage of disease, BMI was not associated with risk for organ-localised prostate cancer (RR=1.18 for ⩾25 *vs* ⩽21.9), although RR tended to increase with increasing BMI for advanced cases (RR=1.38 for ⩾25 *vs* ⩽21.9). In multivariate analysis, the degrees of attenuation in both localised cancer and advanced cancer were similar (data not shown). We also found no association between height and total prostate cancer (*P* for trend=0.45). In advanced cases, the higher categories tended to be associated with increased risk of prostate cancer.

## DISCUSSION

To our knowledge, this is the first prospective study to report an association between prostate cancer and anthropometry in Japanese.

The absence of a consistent association between BMI, height and prostate cancer in the present study is in agreement with previous results from Asia (Severson *et al*, 1988; Hsing *et al*, 2000), but not with others from Western countries (Kaaks *et al*, 2000). The effects of anthropometry therefore seem to differ by country and ethnicity. The differences according to country may be due to ethnic variation in hormonal factors. For example, the hormone level of androstane-3*α*-17*β*-diol glucuronide, an index of 5*α*-reductase activity, and IGF-binding protein-3, which modulates the effect of IGF-1, differs according to ethnicity (Platz *et al*, 1999; Wu *et al*, 2001). A second possibility is that the variation in BMI and height among the present subjects was too small to reveal associations with prostate cancer. It is therefore interesting to contemplate whether an association between BMI or height and prostate cancer would have been detected if more subjects with a greater BMI or height had been enrolled.

We also found slightly stronger associations for advanced than localised disease, although this interpretation was hampered by the small number of cases. Obesity is associated with higher circulating levels of leptin, and leptin has angiogenic activity that correlates with metastasis (Kaaks *et al*, 2000; Ribeiro *et al*, 2004).

In conclusion, our results provide no evidence that BMI and height are associated with the risk of prostate cancer among a relatively lean population. However, they do not rule out the possibility that anthropometry is associated with prostate cancer in advanced cases.

## Figures and Tables

**Table 1:** Relative risk of prostate cancer for body mass index (BMI) and height at baseline: JPHC study, 1990–2003

	**BMI (kg/m^2^)**				
	**⩽21.9**	**22.0–23.4**	**23.5–24.9**	**⩾25.0**	***P* for trend**
*All cases*					
Number of cases	94	66	64	87	
Person-years of follow-up	161 231	116 233	107 654	147 806	
Crude incidence rate^a^	58.3	56.8	59.4	58.9	
Age- and area-adjusted relative risk	1.00	1.05	1.20	1.31	0.057
95% CI	Reference	0.77–1.44	0.87–1.65	0.97–1.76	
Multivariate relative risk^b^	1.00	1.04	1.19	1.24	0.13
95% CI	Reference	0.76–1.43	0.86–1.63	0.92–1.67	
					
*Localised cases*					
Number of cases	53	43	33	50	
Multivariate relative risk^b^	1.00	1.16	1.04	1.18	
95% CI	Reference	0.78–1.74	0.67–1.60	0.79–1.76	0.51
					
*Advanced cases*					
Number of cases	28	16	20	27	
Multivariate relative risk^b^	1.00	0.90	1.33	1.38	0.16
95% CI	Reference	0.49–1.67	0.75–2.37	0.80–2.39	
					

	**Height (cm)**				
	**⩽159**	**160–164**	**164–167**	**⩾168**	***P* for trend**
*All cases*					
Number of cases	80	98	75	58	
Person-years of follow-up	110 069	140 842	127 309	154 704	
Crude incidence rate^a^	72.7	69.6	58.9	37.5	
Age- and area-adjusted relative risk	1.00	1.30	1.30	1.17	0.33
95% CI	Reference	0.97–1.76	0.94–1.80	0.82–1.66	
Multivariate relative risk^c^	1.00	1.27	1.24	1.08	0.45
95% CI	Reference	0.93–1.73	0.88–1.75	0.73–1.59	
					
*Localised cases*					
Number of cases	51	57	46	25	
Multivariate relative risk^c^	1.00	1.13	1.18	0.71	0.48
95%CI	Reference	0.76–1.68	0.76–1.82	0.41–1.22	
					
*Advanced cases*					
Number of cases	21	30	20	20	
Multivariate relative risk^c^	1.00	1.57	1.31	1.51	0.38
95% CI	Reference	0.88–2.81	0.68–2.53	0.74–3.10	

CI=confidence interval; JPHC=Japan Public Health Centre-based Prospective Study.

a Number of prostate cancer cases per 100 000 person-years.

b Adjusted for age, area, smoking status, family history of prostate cancer and marital status.

c Adjusted for age, area, smoking status, family history of prostate cancer, marital status and body weight.

## Appendix A

Members of the JPHC Study Group (principal investigator: S Tsugane): S Tsugane, M Inoue, T Sobue, and T Hanaoka, Research Center for Cancer Prevention and Screening, National Cancer Center, Tokyo; J Ogata, S Baba, T Mannami and A Okayama, National Cardiovascular Center, Suita; K Miyakawa, F Saito, A Koizumi, Y Sano, and I Hashimoto, Iwate Prefectural Ninohe Public Health Center, Ninohe; Y Miyajima, N Suzuki, S Nagasawa and Y Furusugi, Akita Prefectural Yokote Public Health Center, Yokote; H Sanada, Y Hatayama, F Kobayashi, H Uchino, Y Shirai, T Kondo, R Sasaki, Y Watanabe and Y Miyagawa, Nagano Prefectural Saku Public Health Center, Saku; Y Kishimoto, E Takara, T Fukuyama, M Kinjo, M Irei and H Sakiyama, Okinawa Prefectural Chubu Public Health Center, Okinawa; K Imoto, H Yazawa, T Seo, A Seiko, F Ito and F Shoji, Katsushika Public Health Center, Tokyo; A Murata, K Minato, K Motegi and T Fujieda, Ibaraki Prefectural Mito Public Health Center, Mito; K Matsui, T Abe, M Katagiri and M Suzuki, Niigata Prefectural Kashiwazaki and Nagaoka Public Health Center, Kashiwazaki and Nagaoka; M Doi, A Terao and Y Ishikawa, Kochi Prefectural Chuo-higashi Public Health Center, Tosayamada; H Sueta, H Doi, M Urata, N Okamoto and F Ide, Nagasaki Prefectural Kamigoto Public Health Center, Arikawa; H Saldyama, N Onga and H Takaesu, Okinawa Prefectural Miyako Public Health Center, Hirara; F Horii, I Asano, H Yamaguchi, K Aoki, S Maruyama and M Ichii, Osaka Prefectural Suita Public Health Center, Suita; S Matsushima and S Natsukawa, Saku General Hospital, Usuda; M Akabane, Tokyo University of Agriculture, Tokyo; M Konishi and K Okada, Ehime University. Toon; H Iso, Osaka University, Suita; Y Honda and K Yamagishi, Tsukuba University, Tsukuba; H Sugimura, Hamamatsu University, Hamamatsu; Y Tsubono, Tohoku University, Sendai; M Kabuto, National Institute for Environmental Studies, Tsukuba; S Tominaga, Aichi Cancer Center Research Institute, Nagoya; M Iida and W Ajiki, Osaka Medical Center for Cancer and Cardiovascular Disease, Osaka; S Sato, Osaka Medical Center for Health Science and Promotion, Osaka; N Yasuda, Kochi University, Nankoku; S Kono, Kyushu University, Fukuoka; K Suzuki, Research Institute for Brain and Blood Vessels Akita, Akita; Y Takashima, Kyorin University, Mitaka; E Maruyama, Kobe University, Kobe; the late M Yamaguchi, Y Matsumura, S Sasaki and S Watanabe, National Institute of Health and Nutrition, Tokyo; T Kadowaki, Tokyo University, Tokyo; Y Kawaguchi, Tokyo Medical and Dental University, Tokyo; H Shimizu, Sakihae Institute, Gifu.
